# Bone Tissue Engineering: Cell Motility, Vascularization, Micro-Nano Scaffolding, and Remodeling

**DOI:** 10.1155/2014/286978

**Published:** 2014-08-19

**Authors:** Guo-Xian Pei, Yi-Xian Qin, Dietmar Werner Hutmacher, Zhi-Yong Zhang

**Affiliations:** ^1^Department of Orthopaedics, Xijing Hospital, Fourth Military Medical University, Xi'an 710032, China; ^2^Department of Biomedical Engineering, Stony Brook University, Stony Brook, NY 11794-5281, USA; ^3^Regenerative Medicine, Institute of Health and Biomedical Innovation, Queensland University of Technology, Kelvin Grove, QLD 4059, Australia; ^4^National Tissue Engineering Center of China, Shanghai 200240, China

The treatment of large bone defect still remains a major clinical challenge worldwide. Bone tissue engineering (BTE) strategies have been developed and demonstrated great potential to address this ever-pressing clinical need. The success of BTE strategies requires the synergetic efforts from multidisciplinary research fields including stem cell, biomaterial scaffold, and bioactive growth factor. Furthermore, in order to ensure their clinical efficacy, it is essential to promote rapid vascularization and osteogenesis for higher implant survival rate and to apply favorable mechanical stimulation for better bone tissue remodeling.

Reflecting the enormous interest of both our readership and authors in this promising field, we are pleased to present this special issue. This special issue presents a total of 9 papers including research papers and reviews that provide a better understanding of the effects of seed cells, biomaterial scaffolds, bioactive growth factors, mechanical stimulation, and vascularization strategies for the tissue engineered bone (TEB).

Osteogenesis is the basic property and the main purpose of the TEB. And the osteogenesis can be achieved by many methods including seed cells, bioactive growth factors, mechanical stimulation, and bioactive composites. In this special issue, V. V. Meretoja et al. report they enhance the osteogenicity of bioactive composites with biomimetic treatment; Y. Chen et al. report the effects of parathyroid hormone on calcium ions in rat bone marrow mesenchymal stem cells and discover the cellular and molecular mechanism of the parathyroid hormone; Y. X. Qin et al. give a review on the mechanotransduction in musculoskeletal tissue regeneration including the effects of fluid flow, loading, and cellular-molecular pathways and provide a better understanding of the mechanical stimulation for bone tissue remodeling. The early osteogenesis in the TEB is mainly the process of endochondral ossification; Y. Yao et al. report the evaluation of insulin medium or chondrogenic medium on proliferation and chondrogenesis of ATDC5 cells to find the better culture medium for the seed cells.

Based on the important roles of blood supply and innervations on the nutrition, regulation, and metabolism of the human organs and tissues, G. Pei et al. have given the concept of constructing the highly vascularized and neurotized tissue-engineered bone simultaneously for repairing large bone defects according to the theory of biomimetics in the year of 2000 and firstly prove the scientificity of this concept by successfully constructing the vascularized and neurotized tissue-engineered bone in large animal models such as the goat and rhesus. In this special issue, G. Pei et al. review recent microsurgical techniques used to construct the vascularized and neurotized tissue-engineered bone and also introduce their relevant work. S. Fu et al. report the neuropeptide substance P can improve the osteoblastic and angiogenic differentiation of bone marrow stem cells in vitro, and this result also shows the possible relationship between the nerve system and the bone. J. Xiu et al. report the different angiogenic abilities of self-setting calcium phosphate cement scaffolds consisting of different proportions of fibrin glue and optimize the proportion of fibrin glue to get the best vascularization.

The ligament-bone junction of TEB is essential to recover the motor function of the bone defect area when using the TEB for patients. And the silk fibroin is a promising ligament scaffold to achieve this proposal. L. Sun et al. report a novel tissue-engineered ligament-bone junction using the immobilized lentivirus vector bonded on chondroitin sulfate-hyaluronate acid-silk fibroin hybrid scaffold and show the great potentials in future clinical applications. H. Li et al. compare the effect of different thickness of HA-coating on microporous silk scaffolds using alternate soaking technology to determine the best thickness of HA coating to silk scaffolds.

*Guo-Xian Pei* *Yi-Xian Qin* *Dietmar Werner Hutmacher* *Zhi-Yong Zhang*

